# Mineral Contamination from Cemetery Soils: Case Study of Zandfontein Cemetery, South Africa

**DOI:** 10.3390/ijerph9020511

**Published:** 2012-02-07

**Authors:** Cornelia Jonker, Jana Olivier

**Affiliations:** Department of Environmental Sciences, University of South Africa, P.O. Box X6, Florida 1710, South Africa; Email: jana1@mweb.co.za

**Keywords:** minerals, heavy metals, cemetery, coffins, burial load, pollution, soil

## Abstract

The burial of coffins may pose an environmental and health hazard since the metals that are used in coffin-making may corrode or degrade into harmful toxins. These may leach into the surrounding soils and groundwater. Very little research has been conducted world-wide on the mineral contamination potential of cemeteries, and virtually none in South Africa. The aim of the study is to determine whether burial practices affect the mineral content of soils in cemeteries. This was done by comparing the mineral concentrations of soils within the Zandfontein Cemetery in Tshwane (Gauteng, South Africa) to those off-site as well as those in zones with high burial loads with those zones with fewer burials. Twenty three soil samples were collected from various sites on- and off-site and analyzed for 31 minerals using ICP-AES. It was found that mineral concentrations of soils within the Zandfontein Cemetery were considerably higher than those off-site. Soil samples in multiple burials blocks also have elevated metal concentrations. These excess metals are probably of anthropogenic origin associated with burial practices and could pose an environmental and human health hazard. Strict monitoring of water quality in boreholes in the vicinity of the cemetery is recommended.

## 1. Introduction

Agriculture, industry and landfills are commonly believed to be major anthropogenic sources of environmental contamination. Little attention has been given to cemeteries as possible pollution sources. Research conducted on the latter has been limited to examining pollutants emanating from the bodies. However, cemeteries are not only the final resting place to bodies but also to coffins and caskets used for the interment of remains. Indeed, recent studies conducted found the highest contamination arising from cemeteries originated from minerals that are released by burial loads [1]. The minerals that are used in coffin-making may corrode or degrade releasing harmful toxic substances [2]. These may be transported from the graves through seepage and diffuse into surrounding soils. From there they may leach into groundwater and become a potential health risk to the residents in areas surrounding the cemetery [3,4,5,6,7,8]. Most existing cemeteries were sited without thinking about potential risks to the local environment or community [9].

Toxic chemicals that may be released into groundwater include substances that were used in embalming and burial practices in the past as well as varnishes, sealers and preservatives and metal handles and ornaments used on wooden coffins. 

Wood preservatives and paints used in coffin construction contain minerals such as copper naphthalene and ammoniac or chromated copper arsenate (CCA) [2,10]. Besides CCA, ammonium copper quaternary (ACQ) and copper boron azole (CBA) are available on the market [11]. Prior to the 1940s, lead compounds were commonly used as colouring agents in paints [12]. Toxic metals such as manganese, nickel, copper and vanadium were also identified in old paint samples [13]. Currently, many paints still contain lead, mercury, cadmium, and chromium [14,15,16,17]. Arsenic is used as a pigment, a wood preservative and as an anti-fouling ingredient while barium is used as a pigment and a corrosion inhibitor [18,19]. 

Metals are also used for the handles and other ornaments that are attached to the outside of a coffin. The fasteners and coffin ornaments also contain minerals such as zinc and zinc- or copper-alloys, silver or bronze. Often these items are spray painted, vacmetalized, electroplated or a combination of these processes to enhance their aesthetic value [20].

Although wood has traditionally been used in South Africa for the construction of coffins, the price of wood is becoming prohibitive and cheaper materials such as cardboard, plywood, MDF boards, supa-wood, chipboard or pressboard are being used as substitutes [21]. These plywood products contain preservatives that are regulated by Hazard Communication Standards (United States Occupational Safety and Health Administration (OSHA) and may contain chromium and copper. Another recent new development overseas is the use the of light-weight titanium for the construction of coffins [22]. 

The current state of knowledge regarding the contamination loads from cemeteries is limited, with only sparse published information available [9]. One of the few studies conducted on spatial variations of metals content of cemetery soils was that by Spongberg and Becks (2000). This study revealed that metal concentrations of copper, lead, zinc and iron in soils in a cemetery in Ohio in the USA not only differed in from one zone to another within the cemetery, but also differ on- and off-site. To date, no such studies have been conducted in South Africa. 

This article aims to investigate whether the mineral contents of soils in a cemetery are affected by burial practices, and thus by anthropogenic activities. In order to achieve this, the mineral contents of soils within a cemetery were compared with those off-site; and the soil mineral contents of densely “populated” areas of a cemetery with those in areas with fewer burials. Since the burial load may impact directly on the mass of anthropogenically introduced minerals into cemetery soils, the spatial distribution of burials and the burial loads were also determined. 

**Figure 1:**
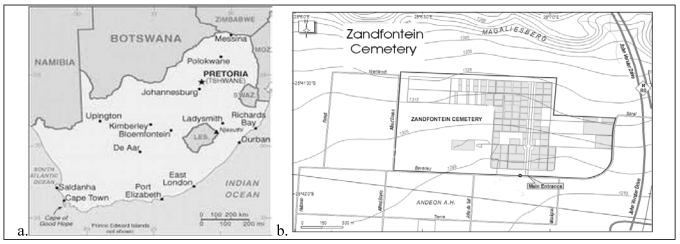
Location and map of study area.

The study was conducted in the city of Tshwane in the province of Gauteng, South Africa. The City of Tshwane Metropolitan area, Pretoria, has a total of 40 cemeteries and one crematorium within the municipal boundary. The Zandfontein Cemetery, the study area, is one of the oldest cemeteries in the City of Tshwane (Pretoria) that is still in operation. Zandfontein Cemetery is located ten kilometres north-west of the city centre on a portion of the farm Zandfontein 318 JR and centres on the following coordinates: S25°41′38.70′′; E28°06′50.86′′ (Figure 1). It is located on the southern slopes of the Magaliesberg. Due to urban encroachment, the cemetery is surrounded by the suburbs of Booysens, Hercules, Kirkney and Andeon L.H. and Lady Selborne.

**Figure 2:**
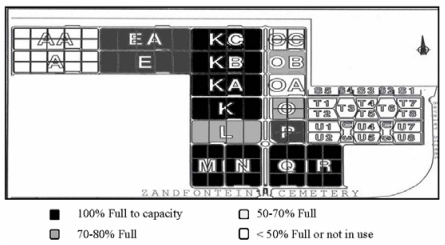
Burial zones in Zandfontein Cemetery.

The cemetery covers an area of about 123.25 ha. It is divided into quadrangular blocks with each block allocated a pre-determined number of burials. The locations of the blocks are shown in Figure 2. At present blocks AA, A, and some plots in S and T have not been used whilst M, N, Q, R, K, KA, KB and KC have reached capacity [22]. Due to the structure of the soils, most blocks were used for single burials (Sandy-loam soils), whereas blocks T and U are used for multiple burials (clayey soils). A total of 60,437 grave plots were used for burials between 1958 and 2010. 

## 2. Methodology

### 2.1. Calculation of Burial Loads

All the data on the burials at Zandfontein Cemetery were obtained from the administrative centre on site. The records on all burials have been noted by hand since 1958 by administrative personnel of the CCTM. Each block or section area in the cemetery has its own record book where the date of burial, particulars of the deceased such as gender and age of the deceased, and grave plot numbers are noted. The number of burials in each of the cemetery zones was obtained from these worksheets. 

A few problems were encountered while attempting to calculate the burial load. Firstly, record-keeping was not always adequate regarding the number of people buried (and hence the number of coffins) in each grave. It is thus difficult to make an accurate estimate of the mass of minerals in any given cemetery, especially in an older, fuller cemetery such as Zandfontein, where grave plots are re-used or where a single grave is used for multiple burials. Moreover, burials take place in different parts of a cemetery at different times and thus exhibit a very large range of spatial and temporal decomposition processes [3]. 

A further shortcoming is that the exact mineral content of each coffin is not known, hence the mass of the mineral content of the burial load could not be determined with any degree of accuracy. However, literature reveals that one coffin handle weighs 300 g [19]. The estimated total metal/mineral mass of the burial load at Zandfontein Cemetery could thus be obtained by multiplying this mass with 6 (handles) and the number of burials. 

### 2.2. Collection and Analysis of Soil Samples

**Figure 3:**
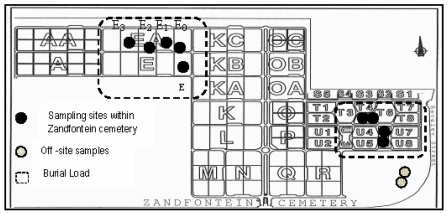
Location of sampling sites.

Soil samples were collected on- and off-site for chemical analysis. The City Council of Tshwane Municipality (CCTM) by-laws on cemeteries stipulate that no person may, unless permitted to do so by the Strategic Executive Officer, disturb the soil in a cemetery [23]. Soil samples were thus only collected from blocks E, EA, T and U, where and whilst contractors for CCTM excavated soil for new grave plots (Figure 3). A total of 23 soil samples were collected from depths ranging between 1 to 2.8 m within the Zandfontein Cemetery. All protocols and safety precautions for collecting possible contaminated soil samples in historical cemeteries were followed, which include wearing a facemask, coverall, booties, and latex gloves. 

To establish the naturally occurring background soil levels for the Zandfontein area, two samples were collected from a nearby off-site area (Figure 3). Samples were collected at one meter depth at each sample point and mixed together into one sample to establish an off-site control sample for the soils inside Zandfontein Cemetery.

One kilogram samples were collected from all sample points and placed in plastic bags. Samples were labelled with date, time, sample I.D, block name and sample depth. Samples were taken to the Agriculture Research Council’s—Institute for Soil Climate and Water (ARC-ISCW) accredited laboratories at Belvedere Street, Pretoria, for analysis. Microwave digestion and Inductively Coupled Plasma-atomic Emission Spectroscopy (ICP-AES) (USEPA Method 6010) were used to analyse 31 micro element concentrations in the soils. Unfortunately, the laboratory did not test for the concentrations of lead and aluminium. 

Means were calculated for each of the minerals in on-site samples. Student’s *t*-test was used to determine whether there is a statistically significant difference between the total mineral content of the soils in different parts of the cemetery. 

## 3. Results and Discussion

The estimated mineral burial mass due to coffins alone is approximately 108,000 kg (6 handles/coffin × 300 g/handle × 60,000 coffins). This mineral mass only accounts for metals that are used in coffin handles and may thus be an underestimation of the total mineral load.

The possibility that cemetery soils are contaminated with toxic minerals was assessed by calculating the ratio of on- to off-site soil mineral content. Table 1 show the mean mineral concentrations and standard deviations of the samples collected within the cemetery and those from off-site samples. The on:off site ratios are also presented. 

**Table 1:** Mean mineral concentrations on- and off- site.

Metal	Mean mineral concentrations on-site (mg/kg) and standard deviations	Mean mineral concentrations off-site (mg/kg) and standard deviations	Approximate ratio of means for on: off-site samples
Li	6.58	2.04	3:1
Be	0.65	0.16	4:1
B	5.99	0.76	8:1
Ti	200.49	26.20	8:1
V	61.59	29.41	2:1
Cr	321.07	76.34	4:1
Mn	430.66	53.44	8:1
Co	20.71	2.56	8:1
Ni	44.63	5.29	8:1
Cu	17.39	3.73	5:1
Zn	7.76	5.93	1:1
As	0.39	0.09	4:1
Se	0.11	0.08	4:1
Rb	10.63	4.48	2:1
Sr	3.06	1.30	2:1
Mo	0.12	0.05	2:1
Cd	0.04	0.02	2:1
Sn	0.15	0.05	3:1
Sb	0.03	0.01	3:1
Te	0.01	0.00	-
Cs	8.78	0.74	11:1
Ba	29.36	6.26	5:1
La	13.21	6.41	2:1
W	0.02	0.00	-
Pt	0.01	0.00	-
Hg	0.02	0.01	2:1
Tl	0.18	0.05	4:1
Pb	26.92	11.84	2:1
Bi	0.10	0.04	3:1
U	0.94	0.38	3:1
Total	1211.6	237.67	5:1

Table 1 indicates that the mean metal concentrations off-site is far less than the on-site metal concentrations. The largest differences in mineral concentrations are those of caesium, boron, manganese, titanium, cobalt and nickel, with ratios exceeding 8:1. The source of the high levels of caesium in the cemetery is not clear since this mineral is not used in coffin construction. The relatively high concentrations of boron, manganese and nickel are more easily explained since these are used either in the metal ornaments or in paints and varnishes on coffins. However, the sources of the relatively high uranium and cobalt loads are not known. Interestingly, Spongberg and Becks (2000) could not explain the presence of high cobalt levels in the Ohio cemetery either. The results at Zandfontein Cemetery for lead correspond to the ratio found in the U.S. [2] but there is relatively more zinc, copper, arsenic, nickel and chrome at Zandfontein. It should also be kept in mind that the Ohio cemetery only had 14,600 graves in comparison to the 60,000+ at Zandfontein. Nevertheless, the results in this study seem to indicate that burial practices do indeed influence the concentration of minerals in cemetery soils. 

Further proof of the anthropogenic origin of soil contamination requires that the areas within the cemetery with high burial loads should have higher mineral concentrations, than those with lower burial loads. 

The approximate number of coffins was obtained by summing the number of graves in the immediate vicinity of the two sets of sample sites *i.e.*, those in blocks E and EA as well as in the adjacent sub-blocks of KA, KB and KC, and those around T_5_ and T_6_ (*i.e.*, T_4–6_ and U_3–8_). The estimated number of burials in the various blocks is shown in Table 2. 

Since the graves in blocks T and U are used for multiple burials, the total number of coffins is higher in these blocks than in the relatively more densely “used” blocks E and EA.

**Table 2:** Estimated number of burials in various blocks of Zandfontein Cemetery (2010).

Blocks	Used grave plots	Blocks	Used grave plots
EA	4442	T_4_	890
E	4375	T_5_	871
KC	4096/4 = 1024	T_6_	1116
KB	4126/4 = 1031	U_3_	671
KA	4126/4= 1031	U_4_	1613
		U_5_	-
		U_6_	367
		U_7_	692
		U_8_	648
Total no graves used	11,903	Total no. graves used	6869
Estimated no burials	**11,903** (single plot burials)	Total no. burials	6869 × 3 (multiple burials) = **20,607**

If the mineral content in the soils is influenced by the burial loads, the mineral content of soil samples collected in the T and U should exceed those in blocks E and EA. This assumption was tested using data obtained for each of the blocks, as shown in Table 3. 

**Table 3:** Mean soils mineral concentrations in various blocks of Zandfontein Cemetery (mg/kg).

mg/kg	Sample points in blocks in Zandfontein									Total
**Metal**	**T_5_**	**T_5_**	**U_6_**	**U_6_**	**EA_O_**	**EA_1_**	**EA_2_**	**EA_3_**	**E**	
**Li**	6.58	6.27	4.73	4.84	4.49	7.45	4.05	8.57	12.27	**59.25**
**Be**	0.84	0.85	0.70	0.76	0.36	0.64	0.52	0.75	0.42	**5.84**
**B**	1.44	1.19	1.97	3.07	0.56	1.04	0.47	34.74	9.47	**53.95**
**Ti**	228.91	467.80	**319.10**	**354.65**	70.86	91.06	135.40	88.30	48.33	**1804.41**
**V**	95.29	92.61	53.66	56.08	39.99	50.58	61.56	61.31	43.20	**554.28**
**Cr**	325.00	363.67	193.93	254.57	234.00	608.45	395.00	363.95	151.03	**2889.6**
**Mn**	1623.6	566.30	499.13	512.33	95.26	109.30	156.64	256.30	57.10	**3875.96**
**Co**	62.06	29.91	22.10	20.77	7.34	9.32	12.19	17.87	4.86	**186.42**
**Ni**	69.98	72.47	47.29	56.87	21.03	39.88	24.81	54.08	15.27	**401.68**
**Cu**	31.14	24.84	18.29	20.18	7.03	13.14	17.92	15.05	8.97	**156.56**
**Zn**	12.47	9.88	8.74	10.15	4.68	5.17	4.47	9.98	4.34	**69.88**
**As**	0.92	0.53	0.37	0.35	0.31	0.11	0.21	0.51	0.20	**3.51**
**Se**	0.14	0.08	0.16	0.14	0.08	0.07	0.14	0.15	0.08	**1.04**
**Rb**	9.25	8.46	10.09	9.05	10.16	14.09	7.84	17.13	9.58	**95.65**
**Sr**	2.83	2.86	2.53	3.08	2.23	2.50	1.31	7.22	2.98	**27.54**
**Mo**	0.22	0.16	0.12	0.10	0.08	0.07	0.15	0.13	0.07	**1.1**
**Cd**	0.05	0.07	0.03	0.06	0.04	0.03	0.02	0.03	0.02	**0.35**
**Sn**	0.20	0.24	0.24	0.22	0.08	0.08	0.11	0.14	0.05	**1.36**
**Sb**	0.04	0.04	0.04	0.03	0.02	0.02	0.03	0.04	0.02	**0.28**
**Te**	0.02	0.02	0.01	0.01	0.01	0.01	0.01	0.01	0.00	**0.1**
**Cs**	1.49	1.53	1.70	1.51	66.24	1.87	1.23	2.16	1.28	**79.01**
**Ba**	95.79	21.52	26.76	29.35	15.93	20.92	13.00	26.40	14.58	**264.25**
**La**	10.52	10.40	14.66	18.88	9.19	13.16	11.80	16.76	13.50	**118.87**
**W**	0.03	0.02	0.03	0.02	0.01	0.01	0.01	0.02	0.01	**0.16**
**Pt**	0.02	0.02	0.01	0.01	0.01	0.00	0.01	0.03	0.01	**0.12**
**Hg**	0.05	0.03	0.04	0.02	0.01	<0.01	0.02	0.03	<0.01	**0.2**
**Tl**	0.48	0.20	0.19	0.19	0.11	0.08	0.11	0.19	0.09	**1.64**
**Pb**	37.47	17.59	17.26	13.09	11.58	17.62	93.94	20.11	13.65	**242.31**
**Bi**	0.16	0.16	0.10	0.10	0.06	0.09	0.11	0.11	0.06	**0.95**
**U**	1.12	1.29	0.81	0.77	0.68	1.02	0.99	1.05	0.72	**8.45**
**Total**	**2618.11**	**1701.01**	**1244.79**	**1371.25**	**602.43**	**1007.78**	**944.07**	**1003.12**	**412.16**	

As expected, Student’s *t*-test shows that there is a significantly higher concentration of minerals in blocks T and U (Mean _T, U_ = 1733.8 mg/kg *vs*. mean _E, EA_ = 627.9; *t* = 3.64, df = 7; α = 0.01), signifying that the cause of contamination could be due to burial practices. The concentration of especially titanium, vanadium, chrome, manganese, cobalt, nickel and zinc are considerably higher in blocks T and U than in E and EA. Contrary to the general trends, the lead content is higher in soils from E and EA than from T and U. Exceptionally high levels of boron, rubidium and strontium were found in soils in EA_3_, lead in EA_2_, chrome in EA_1_ and caesium in EA_0_. The latter cannot be explained.

## 4. Conclusion

Approximately 60,000 coffins have been buried at the Zandfontein Cemetery in Tswane (Pretoria, South Africa). These are estimated to produce a burial load of approximately 108,000 kg minerals. This study was aimed at determining whether this burial load affected the mineral composition of the cemetery soils, thereby causing a potential health risk. 

It was found that the mineral composition of soils within Zandfontein Cemetery was significantly higher than those off-site and that the soils in the zones with the highest burial loads were more contaminated than in the less used parts of the cemetery. This indicates that burial loads have a direct impact on soil-mineral content and thus cemeteries can be regarded as anthropogenic sources of contamination. 

It should be kept in mind that the research did not include the pathogenic or organic releases from gravesites due to burials. It relies on estimations of the amount of metals that are already introduced into the Zandfontein cemetery. Because burials are not carried out in a fixed pattern the results reflect metal contamination from metal deposits that have accumulated over time and not necessarily from metals that have recently been introduced into cemetery soils. Moreover, these results do not necessarily reflect the situation at other cemeteries in Tshwane. The fact that this cemetery is located on the slopes of a mountain may cause leaching of minerals into groundwater and aggravate potential health risks. 

It is recommended that the mineral concentration of groundwater be measured and monitored at boreholes in the surrounding suburbs. Similar studies should be conducted at other cemeteries—not only in Tshwane but countrywide. Such studies will also establish whether cemeteries should be considered to be potential anthropogenic contamination sources—similar to—or even more hazardous than landfill sites.
